# Optimal Power Allocation of Relay Sensor Node Capable of Energy Harvesting in Cooperative Cognitive Radio Network

**DOI:** 10.3390/s17030648

**Published:** 2017-03-21

**Authors:** Pham Ngoc Son, Dongsoo Har, Nam Ik Cho, Hyung Yun Kong

**Affiliations:** 1 Faculty of Electrical and Electronics Engineering, Ho Chi Minh City University of Technology and Education, Ho Chi Minh City 70000, Vietnam; sonpndtvt@hcmute.edu.vn; 2 Cho Chun Shik Graduate School of Green Transportation, KAIST—Korea Advanced Institute of Science & Technology, Daejeon 34141, Korea; dshar@kaist.ac.kr; 3 Department of Electrical and Computer Engineering, Seoul National University, Seoul 08826, Korea; nicho@snu.ac.kr; 4 Department of Electrical and Electronic Engineering, University of Ulsan, Ulsan 44610, Korea; hkong@ulsan.ac.kr

**Keywords:** cooperative communication, energy harvesting, superposition coding, optimal power allocation, relay sensor

## Abstract

A cooperative cognitive radio scheme exploiting primary signals for energy harvesting is proposed. The relay sensor node denoted as the secondary transmitter (ST) harvests energy from the primary signal transmitted from the primary transmitter, and then uses it to transmit power superposed codes of the secrecy signal of the secondary network (SN) and of the primary signal of the primary network (PN). The harvested energy is split into two parts according to a power splitting ratio, one for decoding the primary signal and the other for charging the battery. In power superposition coding, the amount of fractional power allocated to the primary signal is determined by another power allocation parameter (e.g., the power sharing coefficient). Our main concern is to investigate the impact of the two power parameters on the performances of the PN and the SN. Analytical or mathematical expressions of the outage probabilities of the PN and the SN are derived in terms of the power parameters, location of the ST, channel gain, and other system related parameters. A jointly optimal power splitting ratio and power sharing coefficient for achieving target outage probabilities of the PN and the SN, are found using these expressions and validated by simulations.

## 1. Introduction

Cognitive radio (CR), which involves cognitive sensing, has been proposed to increase spectrum utilization of the licensed frequency band [1,2,3]. A licensed primary network (PN) can use the frequency band at any time for signal transmission, whereas the secondary network (SN) senses the frequency band to make opportunistic transmissions. An underlay protocol was also proposed [4,5,6]. With this protocol, the SN can share the frequency band and the operation time with the PN, as long as its signal does not affect the signal of the PN. In [7,8,9,10], the spectrum leasing protocols of the CR networks, in the form of cooperative communication (CC), were investigated. In the CC scheme, the PN leases part of the licensed band to the SN to increase the QoS by having the SN relay the primary signal, and as a reward for relaying the PN signal, the SN is given opportunity to access the licensed band. The decode-and-forward (DF) technique involves a secondary transmitter (ST) capable of sensing and decoding the signal received from the primary transmitter (PT), which forwards the re-coded signal to the primary receiver (PR). On the other hand, the ST of the amplify-and-forward (AF) technique only amplifies and forwards the received signal without use of a complex decoding process. However, unlike the DF technique, the AF technique has a noise amplification problem.

Energy harvesting is considered an effective solution in energy-limited wireless networks, in which it is difficult to replace or recharge the batteries of wireless devices [11,12,13]. Typically, in practice, power splitting circuits connected to the antenna units divide the received radio frequency (RF) signal into two lower-powered RF signals, one signal to charge the battery and the other to process the information received. Unlike with signal splitting, a time switching receiver operates in a time division mode (i.e., the time is divided into two distinct intervals, the first for energy harvesting and the second for information detection [14]). CR networks with energy harvesting capability have been addressed in [15,16,17,18,19]. For the CC schemes in [17,18], in particular, the ST harvests energy from a received signal transmitted by the PT, and acts as a relay for the PN. In [20,21,22,23,24], energy harvesting by sensors in wireless sensor networks is investigated. Sensors are able to harvest energy from human bodies [20,21], neighboring wireless sensors [22,23], and power beacon stations [24]. In [25], energy harvesting from jammers and interference is presented.

Power superposition coding is a multiuser transmission method that intentionally introduces co-channel interference at the transmitter and performs successive interference cancellations at the receivers. The transmitted power of a user code is appropriately adjusted according to geometrical proximity, and multiple codes are superposed in the power domain over the same carrier frequency [26]. In [9], the design and implementation of the encoding and decoding blocks for power superposition coding were addressed. The superposition coding scheme in [10] deals with communications between a pair of users and a pair of base stations.

In this paper, a CC scheme exploiting energy harvesting and power superposition coding is explored, considering a sensor network coexisting with a communication network. The ST of the sensor network acts as a relay for the PT of the communication network, and transmits its own data by power superposition coding. Our scheme, which is operated over two successive phases, is different from the one presented in [27] based on three successive phases. In [27], the second phase of the three successive phases is dedicated for transmission from ST to PR, and the third phase is used for transmission from ST to a secondary receiver (SR). In our scheme, the transmissions from ST to the PR and SR are executed by power superposition coding in the second phase. Unlike the conventional CC scheme [9], the ST in our scheme uses the RF signal transmitted from the PT to charge its internal battery and transmits its own signal and the primary signal, at the same time, by power superposition coding. Therefore, two power parameters are involved with the CC scheme under consideration. The power splitting ratio indicates the fraction of RF power harvested for battery charging, while the power sharing coefficient represents the fraction of power allocated to the primary signal in power superposition coding. Our main concern here is to investigate the impact of the two power parameters on the outage performances (probabilities) of the PN and the SN. To provide a more comprehensive view of system operation, the impact of other system parameters on the outage performances is also examined. Analytical or mathematical expressions of the outage probabilities of the PN and the SN are derived in terms of the power parameters, the location of the ST, the channel gain, and so on. A jointly optimal power splitting ratio and power sharing coefficient able to achieve minimum outage probabilities for the PN and the SN were found using these expressions.

Contributions: The main contributions of this paper are as follows:

(1) Energy harvesting in combination with power superposition coding, both performed by the ST for the CC scheme, is herein considered for the first time. The outage probabilities of the PN and the SN, according to the power splitting ratio and the power sharing coefficient, are assessed by numerical analysis and Monte-Carlo simulation. The relay network presented by Huang et al. [28] also deals with optimal power allocation. However, their work did not involve an SR in the secondary network, whereas ours takes the SR into consideration for optimal power allocation. The optimal power allocation schemes in [29,30] are in relation to relay selection, unlike ours, which employs a single relay sensor to execute power superposition coding.
(2) The jointly optimal power splitting ratio and power sharing coefficient were found using specific analytical or mathematical expressions. In these expressions, the impact of the system parameters (including the two power parameters) on the outage probabilities are evaluated.
(3) The range of the power sharing coefficient that could provide an outage probability of the PN lower than the one obtained by direct transmission from the PT to the PR is identified. Other new findings are presented in the figures in Section 4. The rest of this paper is organized as follows. In Section 2, a system model corresponding to the proposed scheme is described. In Section 3, the analytical or mathematical expressions are derived that will be used to determine the outage probabilities of the PN and the SN according to the power splitting ratio and the power sharing coefficient. Section 4 presents the performance evaluation, according to the two power parameters. Section 5 concludes the paper.

## 2. System Model

### 2.1. System Operation in Two Phases

**Figure 1:**
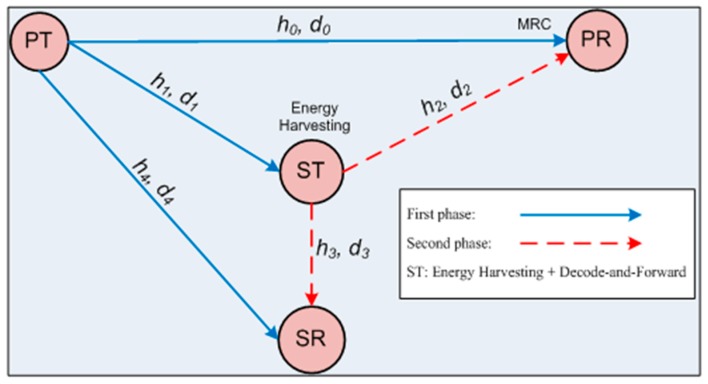
System model of the proposed cooperative communication scheme.

Figure 1 presents the system model, operated over two successive phases. The system model in Figure 1 can be conceived as a heterogeneous sensor network. Cameras and motion sensors (as the PTs) in a sensor network always send surveillance data to a main supervision station (as the PR), using a dedicated frequency band. Temperature change sensors, water level sensors, and humidity sensors (as the STs), using the same frequency band, form another sensor network with an access point (as the SR). This model is described in [31,32]. Our system model assumes that the ST acting as the relay of the PT and as the source of a sensor network is capable of energy harvesting. The devices PT, PR, ST, and SR can be set up with different technologies. If the ST is allowed to help the PT, the ST can change its sensor network configuration by software operations [32].

The system model in [27] is similar to ours with the exceptions that it is operated in three successive phases, and that the ST is not capable of energy harvesting. In the first phase of the system model in Figure 1, the PT multicasts a signal *x_p_*, where $E\left[{\left|x_{p}\right|}^{2}\right]=1,$ and the ST decodes received *x_p_* and combines it with its own signal *x_s_*. In the second phase, the ST multicasts the combined signal to the PR and the SR. It is assumed that the PR is capable of maximal ratio combining (MRC) ([8], (Equation 23); [9]) and that the SR can decode the PT signal in the first phase and cancel it in the second phase. The ST harvests energy from the received signal in the first phase with energy conversion efficiency *η*. Here, *η* (0 < *η* ≤ 1) is defined as the ratio of harvested energy to incident energy and depends on the rectification efficiency and the energy harvesting circuitry of the ST [12,33]. The RF signal received by the ST is split into two signals by the power splitting circuit, according to the power splitting ratio *ρ*, where 0 < *ρ* < 1 [12]. In Figure 1, (*h_i_*, *d_i_*), where 0 ≤ *i* ≤ 4, denotes the Rayleigh block fading channel coefficient, where the channel coefficient is a constant over a phase and varies over every other phase; and the normalized link distance, $d_{i}=D_{i}/D_{0}$, where $D_{i}$ is the distance between two nodes and $D_{0}$ the distance between the PT and the PR, is considered the largest one. The normalized distances are often considered in other papers, such as [12]. The ST can harvest energy from the RF primary signal transmitted by the PT to the PR (e.g., as a downlink transmission). The ST can also periodically transmit its own signal to the SR, together with the relayed primary signal.

The channel gain $g_{i}={\left|h_{i}\right|}^{2}$ is an exponentially distributed random variable (RV) with parameter $\lambda_{i}=d_{i}^{\beta },$ where *β* is the path-loss exponent. Then, the probability density function (pdf) and the cumulative distribution function (CDF) of $g_{i}$ are given by $f_{g_{i}}(x)=\lambda_{i}e^{-\lambda_{i}x},\;x\geq 0$ and $F_{g_{i}}(x)=1-e^{-\lambda_{i}x},$ respectively. The channel gains for data decoding at the PR and the SR are assumed to be obtained by the medium access control (MAC) protocol specified in [34]. The antenna gains for signal transmission and reception at PT, PR, ST, and SR are set to ‘1’.

The received signals at the PR, ST, and SR in the first phase are obtained, respectively, as

(1) $$y_{PT-PR}=\sqrt{P}\times h_{0}\times x_{p}+n_{PR}$$

(2) $$y_{PT-ST}=\sqrt{P}\times h_{1}\times x_{p}+n_{ST}$$

(3) $$y_{PT-SR}=\sqrt{P}\times h_{4}\times x_{p}+n_{SR}$$

where *P* is the transmit power of the PT, and $n_{PR},\;n_{ST}$, and $n_{SR}$ denote additive white Gaussian noise (AWGN) values of the same variance *N*_0_ at the PR, ST, and SR, respectively.

The power splitting circuit of the ST splits the received signal $y_{PT-ST}$ into two lower power signals $\sqrt{\rho }\times y_{PT-ST}$ and $\sqrt{\left(1-\rho \right)}\times y_{PT-ST}$, where *ρ* (0 < *ρ* < 1) is the power splitting ratio. The fraction $\sqrt{\rho }\times y_{PT-ST}$ is used for charging the battery and the remaining $\sqrt{\left(1-\rho \right)}\times y_{PT-ST}$ is used for decoding of *x_p_*. Specifically, the received signal $y_{PT-ST}^{C}$ at the ST used for charging, is expressed as

(4) $$y_{PT-ST}^{C}=\sqrt{\rho }\times y_{PT-ST}=\sqrt{\rho P}\times h_{1}\times x_{p}+\sqrt{\rho }\times n_{ST}$$

Therefore, the energy used for charging during the first phase (with time duration *T*_1_) can be described as

(5) $$E_{PT-ST}^{C}=\rho P{\left|h_{1}\right|}^{2}\eta T_{1}=\rho \eta PT_{1}g_{1}$$

The noise energy carried by $\sqrt{\rho }\times n_{ST}$ in (4) is assumed to be comparatively negligible, so it can be omitted for $E_{PT-ST}^{C}$ in (5) [11,12].

The received signal $y_{PT-ST}^{d}$ at the ST to be consumed for decoding in the first phase is given as

(6) $$y_{PT-ST}^{d}=\sqrt{\left(1-\rho \right)}\times y_{PT-ST}=\sqrt{\left(1-\rho \right)P}\times h_{1}\times x_{p}+\sqrt{\left(1-\rho \right)}\times n_{ST}$$

Considering the additional noise generated by the RF-to-baseband conversion units (RFBCUs) for sampling [12] at the PR, ST, and SR; Equations (1), (6) and (3) are modified as

(7) $$y_{PT-PR}=\sqrt{P}\times h_{0}\times x_{p}+n_{PR}+n_{PR}^{c}$$

(8) $$y_{PT-ST}^{d}=\sqrt{\left(1-\rho \right)P}\times h_{1}\times x_{p}+\sqrt{\left(1-\rho \right)}\times n_{ST}+n_{ST}^{c}$$

(9) $$y_{PT-SR}=\sqrt{P}\times h_{4}\times x_{p}+n_{SR}+n_{SR}^{c}$$

where $n_{PR}^{c}$, $n_{ST}^{c}$, and $n_{SR}^{c}$ denote the AWGNs due to the RFBCUs at the PR, ST, and SR, respectively, with the same variance *µN*_0_, *µ* > 0. We can assume that all wireless nodes have the same structure, so that all AWGNs are statistically identical.

### 2.2. SNRs and SINRs of Signals

The SNRs (signal-to-noise ratios) of the signals received by the PR, ST, and SR, taking into account the additional noise due to conversion by the RFBCUs, are obtained from Equations (7)–(9) as

(10) $$\gamma_{PR}=\frac{P{\left|h_{0}\right|}^{2}}{N_{0}+\mu N_{0}}=\frac{\gamma g_{0}}{1+\mu }$$

(11) $$\gamma_{ST}=\frac{\left(1-\rho \right)P{\left|h_{1}\right|}^{2}}{\left(1-\rho \right)N_{0}+\mu N_{0}}=\frac{\left(1-\rho \right)\gamma g_{1}}{1+\mu -\rho }$$

(12) $$\gamma_{SR}=\frac{P{\left|h_{4}\right|}^{2}}{N_{0}+\mu N_{0}}=\frac{\gamma g_{4}}{1+\mu }$$

where *γ* = *P/N*_0_ is defined as the transmit SNR.

Because the decode-and-forward type CC scheme is our concern, the ST decodes the *x_p_* before forwarding it. In the first phase, the ST combines the decoded *x_p_* with its own signal *x_s_* by superposition coding as follows [10]

(13) $$x_{c}=\sqrt{\alpha P_{ST}}\times x_{p}+\sqrt{\left(1-\alpha \right)P_{ST}}\times x_{s}$$

where *x_c_* is the combined signal. The *P_ST_* is the total transmit power of the ST and it is divided into two lower-power components $\alpha P_{ST}$ and $\left(1-\alpha \right)P_{ST}$, where *α* (0 < *α* < 1) is the power sharing coefficient. Here, $\alpha P_{ST}$ is assigned to the data *x_p_* to help the PT in forwarding it to the PR, and $\left(1-\alpha \right)P_{ST}$ is used to transmit the data *x_s_* of the ST to the SR.

In the second phase, the ST multicasts the data *x_c_* to the PR and the SR, with the transmit power *P_ST_* in (13), which can be provided from the harvested energy $E_{PT-ST}^{h}$ in (5), along with a small portion of the energy stored in the battery of the ST. Let *Ψ* be the fractional constant and *Ψ* *P* be the transmit power provided by the battery. Then, the transmit power *P_ST_* in (13) is obtained from (5) as follows

(14) $$P_{ST}=\frac{E_{PT-ST}^{C}}{T_{2}}+\psi P=\left(\frac{T_{1}}{T_{2}}\right)\rho \eta g_{1}P+\psi P$$

where *T*_2_ is the time duration of the second phase. Assuming the same data transmission rate from the PT and the ST, *T*_1_ is set equal to *T*_2_. When ψ=0, *P_ST_* in (14) is solely provided by the energy harvested from the PT.

The signals received by the PR and the SR in the second phase from the ST can be expressed as

(15) $$y_{ST-PR}=x_{c}\times h_{2}+n_{PR}=\left(\sqrt{\alpha P_{ST}}\times x_{p}+\sqrt{\left(1-\alpha \right)P_{ST}}\times x_{s}\right)\times h_{2}+n_{PR}$$

(16) $$y_{ST-SR}=x_{c}\times h_{3}+n_{SR}=\left(\sqrt{\alpha P_{ST}}\times x_{p}+\sqrt{\left(1-\alpha \right)P_{ST}}\times x_{s}\right)\times h_{3}+n_{SR}$$

Considering the RFBCUs, (15) and (16) can be modified as

(17) $$\begin{array}{ll} y_{ST-PR} & =\left(\sqrt{\alpha P_{ST}}\times x_{p}+\sqrt{\left(1-\alpha \right)P_{ST}}\times x_{s}\right)\times h_{2}+n_{PR}+n_{PR}^{c}\\ & \;=\underset{\mathrm{desired}\;\mathrm{component}}{\underset{︸}{\sqrt{\alpha P_{ST}}\times x_{p}\times h_{2}}}+\underset{\mathrm{interference}\;\mathrm{component}}{\underset{︸}{\sqrt{\left(1-\alpha \right)P_{ST}}\times x_{s}\times h_{2}}}+n_{PR}+n_{PR}^{c} \end{array}$$

(18) $$\begin{array}{ll} y_{ST-SR} & =\left(\sqrt{\alpha P_{ST}}x_{p}+\sqrt{\left(1-\alpha \right)P_{ST}}x_{s}\right)h_{3}+n_{SR}+n_{SR}^{c}\\ & \;=\underset{\mathrm{desired}\;\mathrm{component}}{\underset{︸}{\sqrt{\left(1-\alpha \right)P_{ST}}x_{s}h_{3}}}+\underset{\mathrm{interference}\;\mathrm{component}}{\underset{︸}{\sqrt{\alpha P_{ST}}x_{p}h_{3}}}+n_{SR}+n_{SR}^{c} \end{array}$$

The PR adopts the MRC technique to combine the two signals, one received from the PT in the first phase, and the other from the ST in the second phase, in order to decode *x_p_*. Thus, the signal-to-interference-plus-noise ratio (SINR) of the signal received at the PR can, in terms of the two power parameters and other system parameters, from (10) and (17) ([8], Equation (23)) be obtained as

(19) $$\begin{array}{ll} \gamma_{PR,SINR} & =\gamma_{PR}+\frac{\alpha P_{ST}|h_{2}{|}^{2}}{\left(1-\alpha \right)P_{ST}|h_{2}{|}^{2}+N_{0}+\mu N_{0}}\\ & =\gamma_{PR}+\frac{\alpha \gamma \left(\rho \eta g_{1}+\psi \right)g_{2}}{\left(1-\alpha \right)\gamma \left(\rho \eta g_{1}+\psi \right)g_{2}+\left(1+\mu \right)} \end{array}$$

The SR receives *x_p_* in the first phase as a part of $y_{PT-SR}$ in (9) and uses this *x_p_* to cancel the interference component in (18) to decode the desired *x_s_*. Hence, there are two cases for decoding the data *x_p_* with the received signal in (9).

Case 1: When the SR is unsuccessful in decoding the *x_p_*, the SINR $\gamma_{SR,SINR}^{U}$ at the SR from (18) becomes

(20) $$\gamma_{SR,SINR}^{U}=\frac{\left(1-\alpha \right)P_{ST}|h_{3}{|}^{2}}{\alpha P_{ST}|h_{3}{|}^{2}+N_{0}+\mu N_{0}}=\frac{\left(1-\alpha \right)\gamma \left(\rho \eta g_{1}+\psi \right)g_{3}}{\alpha \gamma \left(\rho \eta g_{1}+\psi \right)g_{3}+\left(1+\mu \right)}$$

Case 2: When the SR is successful in decoding the *x_p_*, the SR can cancel the interference component *x_p_* in (18) and the signal at the SR after cancellation becomes $y_{ST-SR}=\sqrt{\left(1-\alpha \right)P_{ST}}\times x_{s}\times h_{3}+n_{SR}+n_{SR}^{c}$, so the SINR $\gamma_{SR,SINR}^{S}$ can be determined by

(21) $$\gamma_{SR,SINR}^{S}=\frac{\left(1-\alpha \right)P_{ST}|h_{3}{|}^{2}}{N_{0}+\mu N_{0}}=\frac{\left(1-\alpha \right)\gamma \left(\rho \eta g_{1}+\psi \right)g_{3}}{1+\mu }$$

## 3. Outage Probability Analysis

The outage probability of the PN (or SN) is defined as the probability that the achievable data rate is less than the target data rate due to SNR or SINR lower than a given threshold, at the PR (or SR).

### 3.1. Outage Probability of PN

Let *R_T_*, *R_APR_*, and *R_AST_* be the target primary data rate, achievable data rate at the PR, and achievable data rate at the ST, respectively, all in the first phase. Also, let *R_MRC_* be the achievable data rate at the PR in the second phase. Then, the outage of the PN occurs when (1) *R_AST_* < *R_T_* and *R_APR_* < *R_T_* in the first phase, or (2) *R_MRC_* < *R_T_* in the second phase, when $R_{AST}\geq R_{T}$. This can be described mathematically by

(22) $$P_{PN}^{cc}=\underset{\mathrm{Pr}1}{\underset{︸}{\mathrm{Pr}\left[R_{AST}<R_{T},\;R_{APR}<R_{T}\right]}}+\underset{\mathrm{Pr}2}{\underset{︸}{\mathrm{Pr}\left[R_{AST}\geq R_{T},\;R_{MRC}<R_{T}\right]}}$$

The jointly optimal power splitting ratio and the power sharing coefficient to minimize $P_{PN}^{cc}$ in Equation (22) (while the other system parameters are fixed) can be expressed as

(23) $$(\rho_{PN,jopt}\;\alpha_{PN,jopt})=\underset{\left(\rho \;\alpha \right)}{\arg\min}P_{PN}^{CC}$$

subject to (i) 0 < *ρ* < 1, and (ii) 0 < *α* < 1.

In (23), “*jopt*” in the subscripts of *ρ* and *α* indicates the jointly optimal values of *ρ* and *α* to minimize $P_{PN}^{cc}$; whereas, $\rho_{PN,opt}$, which will appear in Section 4 for the performance evaluation, denotes the marginally optimal value of *ρ* for a given *α* (or other system parameter). Whenever necessary in Section 4, conditions for the marginally optimal value of *ρ* are explicitly indicated.

From (10), (11) and (19), the *R_APR_*, *R_AST_*, and *R_MRC_* can be shown as

(24) $$R_{APR}=\frac{1}{2}\times \log_{2}\left(1+\gamma_{PR}\right)=\frac{1}{2}\times \log_{2}\left(1+\frac{\gamma g_{0}}{1+\mu }\right)$$

(25) $$R_{AST}=\frac{1}{2}\times \log_{2}\left(1+\gamma_{ST}\right)=\frac{1}{2}\log_{2}\left(1+\frac{\left(1-\rho \right)\gamma g_{1}}{1+\mu -\rho }\right)$$

(26) $$\begin{array}{ll} R_{MRC} & =\frac{1}{2}\times \log_{2}\left(1+\gamma_{PR,SINR}\right)\\ & =\frac{1}{2}\times \log_{2}\left(1+\gamma_{PR}+\frac{\alpha \gamma \left(\rho \eta g_{1}+\psi \right)g_{2}}{\left(1-\alpha \right)\gamma \left(\rho \eta g_{1}+\psi \right)g_{2}+\left(1+\mu \right)}\right) \end{array}$$

where the factor 1/2 indicates that the CC scheme is operated in two phases.

Substituting (24) for *R_APR_*, (25) for *R_AST_*, and (26) for *R_MRC_*, Pr1 in (22) is obtained as

(27) $$\begin{array}{ll} \mathrm{Pr}1 & =\mathrm{Pr}\left[\frac{1}{2}\times \log_{2}\left(1+\frac{\gamma g_{0}}{1+\mu }\right)<R_{T},\;\frac{1}{2}\times \log_{2}\left(1+\frac{\left(1-\rho \right)\gamma g_{1}}{1+\mu -\rho }\right)<R_{T}\right]\\ & =\mathrm{Pr}\left[g_{0}<\underset{\theta_{0}}{\underset{︸}{\frac{\left(2^{2R_{T}}-1\right)\left(1+\mu \right)}{\gamma }}},\;g_{1}<\underset{\theta_{1}}{\underset{︸}{\frac{\left(2^{2R_{T}}-1\right)\left(1+\mu -\rho \right)}{\gamma \left(1-\rho \right)}}}\right]\\ & =F_{g_{0}}\left(\theta_{0}\right)\times F_{g_{1}}\left(\theta_{1}\right)=\left(1-e^{-\lambda_{0}\theta_{0}}\right)\times \left(1-e^{-\lambda_{1}\theta_{1}}\right) \end{array}$$

Pr2 in (22) is also obtained as

(28) $$\begin{array}{ll} \mathrm{Pr}2 & =\mathrm{Pr}\left[\;\frac{1}{2}\times \log_{2}\left(1+\frac{\left(1-\rho \right)\gamma g_{1}}{1+\mu -\rho }\right)\geq R_{T},\text{ ​}\frac{1}{2}\times \log_{2}\left(1+\gamma_{PR}+\frac{\alpha \gamma \left(\rho \eta g_{1}+\psi \right)g_{2}}{\left(1-\alpha \right)\gamma \left(\rho \eta g_{1}+\psi \right)g_{2}+\left(1+\mu \right)}\right)<R_{T}\right]\\ & =\mathrm{Pr}\left[g_{1}\geq \theta_{1},\;\gamma_{PR}+\frac{\alpha \gamma \left(\rho \eta g_{1}+\psi \right)g_{2}}{\left(1-\alpha \right)\gamma \left(\rho \eta g_{1}+\psi \right)g_{2}+\left(1+\mu \right)}<\underset{\tau }{\underset{︸}{2^{2R_{T}}-1}}\right] \end{array}$$

Let $X\overset{\Delta }{=}\rho \eta g_{1}+\psi$ and $Y\overset{\Delta }{=}\alpha \gamma Xg_{2}/[\left(1-\alpha \right)\gamma Xg_{2}+\left(1+\mu \right)].$ Then, the CDF and pdf of RV *X* are given as

(29) $$F_{X}\left(x\right)=\mathrm{Pr}\left[\rho \eta g_{1}+\psi <x\right]=\mathrm{Pr}\left[g_{1}<\frac{x-\psi }{\rho \eta }\right]=\left\{ \begin{array}{l} 0\;,\;x\leq \psi \\ 1-e^{-\lambda_{1}\left(x-\psi \right)/\left(\rho \eta \right)}\;,\;x>\psi \end{array}\right.$$

(30) $$f_{X}\left(x\right)=\frac{\partial F_{X}\left(x\right)}{\partial x}=\left\{ \begin{array}{l} 0\;,\;x\leq \psi \\ \frac{\lambda_{1}}{\rho \eta }e^{-\lambda_{1}\left(\frac{x-\psi }{\rho \eta }\right)}\;,\;x>\psi \end{array}\right.$$

From (10), the CDF and pdf of RV $\gamma_{PR}$ are obtained as

(31) $$F_{\gamma_{PR}}\left(z\right)=\mathrm{Pr}\left[\frac{\gamma g_{0}}{1+\mu }<z\right]=F_{g_{0}}\left(\frac{\left(1+\mu \right)z}{\gamma }\right)=\left\{ \begin{array}{ll} 1-e^{-\frac{\lambda_{0}\left(1+\mu \right)z}{\gamma }} & ,\;z\geq 0\\ 0 & ,\;z<0 \end{array}\right.$$

(32) $$f_{\gamma_{PR}}\left(z\right)=\frac{\partial F_{\gamma_{PR}}\left(z\right)}{\partial z}=\left\{ \begin{array}{ll} \frac{\lambda_{0}\left(1+\mu \right)}{\gamma }e^{-\frac{\lambda_{0}\left(1+\mu \right)z}{\gamma }} & ,\;z\geq 0\\ 0 & ,\;z<0 \end{array}\right.$$

To obtain Pr2 in (28), a lemma is considered.

**Lemma** **1.** *The following expression is valid for the joint probability of RVs Y and g*_1_.

Case 1: For y≥α/(1−α), where *α* is the power sharing coefficient, the joint probability of *Y* and *g*_1_ from (28) is given by

(33) $$\mathrm{Pr}\left[\left(\underset{Y}{\underset{︸}{\frac{\alpha \gamma \left(\rho \eta g_{1}+\psi \right)g_{2}}{\left(1-\alpha \right)\gamma \left(\rho \eta g_{1}+\psi \right)g_{2}+\left(1+\mu \right)}}}<y\right),g_{1}\geq \theta_{1}\right]=e^{-\lambda_{1}\theta_{1}}$$

Case 2: For y<α/(1−α), the joint probability of *Y* and *g*_1_ is obtained by

(34) $$\begin{array}{ll} \mathrm{Pr}\left[Y<y,\;g_{1}\geq \theta_{1}\right]= & e^{-\lambda_{1}\theta_{1}}-2e^{\lambda_{1}\psi /\left(\rho \eta \right)}\sqrt{\frac{\lambda_{1}\lambda_{2}\phi \left(y\right)}{\rho \eta }}\;K_{1}\left(2\sqrt{\frac{\lambda_{1}\lambda_{2}\phi \left(y\right)}{\rho \eta }}\right)\\ & +\frac{\lambda_{1}}{\rho \eta }e^{\lambda_{1}\psi /\left(\rho \eta \right)}\int_{0}^{\rho \eta \theta_{1}+\psi }e^{\frac{-\lambda_{1}t}{\rho \eta }-\frac{\lambda_{2}\phi \left(y\right)}{t}}dt \end{array}$$

In (33) and (34), the term *K*_1_(·) is the first order modified Bessel function of the second kind ([35], Equation (8.432.6)) and the function *φ*(*y*) is defined as

(35) $$\phi \left(y\right)=\frac{\left(1+\mu \right)y}{\gamma \left[\alpha -\left(1-\alpha \right)y\right]}$$

**Proof.**  The proof is in Appendix A. ☐

From Lemma 1, Pr2 in (28) can be expressed as

(36) $$\begin{array}{ll} \mathrm{Pr}2 & =\mathrm{Pr}\left[X\geq \rho \eta \theta_{1}+\psi ,\;\gamma_{PR}+Y<\tau \right]\\ & =\int_{0}^{\tau }f_{\gamma_{PR}}\left(w\right)\times \mathrm{Pr}\left[X\geq \rho \eta \theta_{1}+\psi ,\;Y<(\tau -w)\right]dw \end{array}$$

**Theorem** **1.** *The following expressions show how Pr2 depends on* τ.

Case 1: For τ<α/(1−α), Pr2 in (36) is given by

(37) $$\begin{array}{ll} \mathrm{Pr}2= & e^{-\lambda_{1}\theta_{1}}\left(1-e^{-\lambda_{0}\left(1+\mu \right)\tau /\gamma }\right)-\frac{2\lambda_{0}\left(1+\mu \right)}{\gamma }\sqrt{\frac{\lambda_{1}\lambda_{2}\left(1+\mu \right)}{\rho \eta \gamma \left(1-\alpha \right)}}\times e^{\lambda_{1}\psi /\left(\rho \eta \right)-\lambda_{0}\left(1+\mu \right)\tau /\gamma }\\ & \times \int_{0}^{\tau }\sqrt{\frac{x}{\alpha /\left(1-\alpha \right)-x}}\times K_{1}\left(2\sqrt{\frac{\lambda_{1}\lambda_{2}\left(1+\mu \right)}{\rho \eta \gamma \left(1-\alpha \right)}}\times \sqrt{\frac{x}{\alpha /\left(1-\alpha \right)-x}}\right)\times e^{\lambda_{0}\left(1+\mu \right)x/\gamma }dx\\ & +\frac{\lambda_{0}\lambda_{1}\left(1+\mu \right)}{\rho \eta \gamma }\times e^{\lambda_{1}\psi /\left(\rho \eta \right)-\lambda_{0}\left(1+\mu \right)\tau /\gamma }\int_{0}^{\tau }\int_{0}^{\rho \eta \theta_{1}+\psi }e^{\lambda_{0}\left(1+\mu \right)x/\gamma -\lambda_{1}t/\left(\rho \eta \right)-\frac{\lambda_{2}\left(1+\mu \right)x}{\gamma \left(\alpha /\left(1-\alpha \right)-x\right)\left(1-\alpha \right)t}}dtdx \end{array}$$

Case 2: For τ≥α/(1−α) Pr2 in (36) is obtained as

(38) $$\begin{array}{ll} \mathrm{Pr}2= & e^{-\lambda_{1}\theta_{1}}\left(1-e^{-\lambda_{0}\left(1+\mu \right)\tau /\gamma }\right)\;-\frac{2\lambda_{0}\left(1+\mu \right)}{\gamma }\sqrt{\frac{\lambda_{1}\lambda_{2}\left(1+\mu \right)}{\rho \eta \gamma \left(1-\alpha \right)}}\times e^{\lambda_{1}\psi /\left(\rho \eta \right)-\lambda_{0}\left(1+\mu \right)\tau /\gamma }\\ & \times \int_{0}^{\alpha /\left(1-\alpha \right)}\sqrt{\frac{x}{\alpha /\left(1-\alpha \right)-x}}\times K_{1}\left(2\sqrt{\frac{\lambda_{1}\lambda_{2}\left(1+\mu \right)}{\rho \eta \gamma \left(1-\alpha \right)}}\times \sqrt{\frac{x}{\alpha /\left(1-\alpha \right)-x}}\right)\times e^{\lambda_{0}\left(1+\mu \right)x/\gamma }dx\\ & +\frac{\lambda_{0}\lambda_{1}\left(1+\mu \right)}{\rho \eta \gamma }\times e^{\lambda_{1}\;\psi /\left(\rho \eta \right)-\lambda_{0}\left(1+\mu \right)\tau /\gamma }\int_{0}^{\alpha /\left(1-\alpha \right)}\int_{0}^{\rho \eta \theta_{1}+\psi }e^{\lambda_{0}\left(1+\mu \right)x/\gamma -\lambda_{1}t/\left(\rho \eta \right)-\frac{\lambda_{2}\left(1+\mu \right)x}{\gamma \left(\alpha /\left(1-\alpha \right)-x\right)\left(1-\alpha \right)t}}dtdx \end{array}$$

**Proof.**  The proof is shown in Appendix B. ☐

From Pr1 in (27) and Pr2 in (37) or (38), the outage probability of the PN in the CC scheme is obtained. Pr1 is obtained as an analytic function, whereas Pr2 in (37) or (38) involves single and double integrals of complex expressions.

For comparison, the outage probability of the PN in the direct transmission (DT) scheme is evaluated. Using the DT scheme, the PT directly transmits *x_p_* to the PR without relaying it via the ST. For a fair comparison, the PT transmits the signal *x_p_* twice over two consecutive phases. At the PR, the MRC technique is used to combine the two received signals involving *x_p_*, to increase the received SNR and improve the decoding performance. From (7), the SNR at the PR after two consecutive phases is obtained as

(39) $$\gamma_{PR}^{DT}=\gamma_{PR,1}+\gamma_{PR,2}=\frac{\gamma }{1+\mu }\left(g_{01}+g_{02}\right)$$

where *g*_01_ and *g*_02_ are the channel gains of link PT–PR in the first phase and in the second phase, respectively. The values of *g*_01_ and *g*_02_ are reasonably assumed to be independent exponential RVs with the same parameter $\lambda_{0}$. The CDF of $\gamma_{PR}^{DT}$ is expressed as

(40) $$\begin{array}{ll} F_{\gamma_{PR}^{DT}}(x) & =\mathrm{Pr}\left[\gamma_{PR}^{DT}<x\right]=\mathrm{Pr}\left[\frac{\gamma \left(g_{01}+g_{02}\right)}{1+\mu }<x\right]=\mathrm{Pr}\left[g_{02}<\frac{\left(1+\mu \right)x}{\gamma }-g_{01}\right]\\ & =\int_{0}^{\left(1+\mu \right)x/\gamma }f_{g_{01}}\left(t\right)\times F_{g_{02}}\left(\left(1+\mu \right)x/\gamma -t\right)dt\\ & =1-\left(1+\lambda_{0}\left(1+\mu \right)x/\gamma \right)e^{-\lambda_{0}\left(1+\mu \right)x/\gamma } \end{array}$$

where $f_{g_{01}}(t)=\lambda_{0}e^{-\lambda_{0}t}$ and $F_{g_{02}}(t)=1-e^{-\lambda_{0}t}$ are the pdf of *g*_01_ and the CDF of *g*_02_, respectively, and $F_{g_{02}}(x\left(1+\mu \right)/\gamma -t)=1-e^{-\lambda_{0}\left(x\left(1+\mu \right)/\gamma -t\right)}.$

From (40) and the definition of τ in (28), the outage probability of the DT scheme is given as

(41) $$\begin{array}{ll} P_{out}^{DT} & =\mathrm{Pr}\left[\frac{1}{2}\times \log_{2}\left(1+\gamma_{PR}^{DT}\right)<R_{T}\right]=F_{\gamma_{PR}^{DT}}(\tau )\\ & =1-\left(1+\lambda_{0}\left(1+\mu \right)\tau /\gamma \right)\times e^{-\lambda_{0}\left(1+\mu \right)\tau /\gamma } \end{array}$$

### 3.2. Outage Probability of the SN

The outage probability of the SN in the CC scheme is considered for three different cases. In the first case, the ST does not successfully decode the value of *x_p_* of the PT in the first phase. In the second and third cases, the ST successfully decodes the value of *x_p_* of the PT in the first phase, and the achievable data rate at the SR is less than the target rate *R_s_* with or without interference cancellation. From these considerations, the outage probability of the SN can be expressed as

(42) $$P_{SN}^{cc}=\underset{\begin{array}{c} \mathrm{ST}\;\mathrm{does}\;\mathrm{not}\;\\ \mathrm{decode}\;x_{P} \end{array}}{\underset{︸}{\mathrm{Pr}\left[R_{AST}<R_{T}\right]}}+\underset{\begin{array}{l} \mathrm{ST}\;\mathrm{decodes}\;x_{P}\;and\;SR\;\mathrm{does}\;\mathrm{not}\;\mathrm{decode}\;x_{s}\;\\ \mathrm{without}\;x_{p}\;\mathrm{cancel}\;\mathrm{lation}\; \end{array}}{\underset{︸}{\mathrm{Pr}\left[R_{AST}\geq R_{T},R_{ASR}<R_{T},\;R_{ASR2}^{wo}<R_{s}\right]}}+\underset{\begin{array}{l} \mathrm{ST}\;\mathrm{decodes}\;x_{P}\;and\;SR\;\mathrm{does}\;\mathrm{not}\;\mathrm{decode}\;x_{s}\;\\ \mathrm{with}\;x_{p}\;\mathrm{cancellation}\; \end{array}}{\underset{︸}{\mathrm{Pr}\left[R_{AST}\geq R_{T},\;R_{ASR}\geq R_{T},R_{ASR}^{w}<R_{s}\right]}}$$

where *R_ASR_*, $R_{ASR2}^{wo}$, and $R_{ASR2}^{w}$ are the achievable data rates at the SR in the first phase, at the SR in the second phase without *x_p_* cancellation, and at the SR in the second phase with *x_p_* cancellation, respectively.

From (12), (20) and (21), the achievable data rates *R_ASR_*, $R_{ASR2}^{wo}$, and $R_{ASR2}^{w}$ are expressed as

(43) $$R_{ASR}=\frac{1}{2}\times \log_{2}\left(1+\gamma_{SR}\right)=\frac{1}{2}\times \log_{2}\left(1+\frac{\gamma g_{4}}{1+\mu }\right)$$

(44) $$R_{ASR2}^{wo}=\frac{1}{2}\times \log_{2}\left(1+\gamma_{SR,SINR}^{U}\right)=\frac{1}{2}\times \log_{2}\left(1+\frac{\left(1-\alpha \right)\gamma \left(\rho \eta g_{1}+\psi \right)g_{3}}{\alpha \gamma \left(\rho \eta g_{1}+\psi \right)g_{3}+\left(1+\mu \right)}\right)$$

(45) $$R_{ASR2}^{w}=\frac{1}{2}\times \log_{2}\left(1+\gamma_{SR,SINR}^{S}\right)=\frac{1}{2}\times \log_{2}\left(1+\frac{\left(1-\alpha \right)\gamma \left(\rho \eta g_{1}+\psi \right)g_{3}}{1+\mu }\right)$$

Substituting (25), (43), (44) and (45) into (42), the outage probability $P_{SN}^{cc}$ can be rewritten as

(46) $$\begin{array}{ll} P_{SN}^{cc} & =\mathrm{Pr}\left[g_{1}<\theta_{1}\right]+\mathrm{Pr}\left[g_{1}\geq \theta_{1},\;g_{4}<\theta_{0},\frac{\left(1-\alpha \right)\gamma \left(\rho \eta g_{1}+\psi \right)g_{3}}{\alpha \gamma \left(\rho \eta g_{1}+\psi \right)g_{3}+\left(1+\mu \right)}<\underset{\upsilon }{\underset{︸}{2^{2R_{s}}-1}}\text{​}\right]\\ & +\mathrm{Pr}\left[g_{1}\geq \theta_{1},\;g_{4}\geq \theta_{0},\frac{\left(1-\alpha \right)\gamma \left(\rho \eta g_{1}+\psi \right)g_{3}}{1+\mu }<\upsilon \text{​}\right]\\ & =F_{g_{1}}\left(g_{1}\right)+F_{g_{4}}\left(g_{0}\right)\times \mathrm{Pr}\left[g_{1}\geq \theta_{1},\frac{\left(1-\alpha \right)\gamma Xg_{3}}{\alpha \gamma Xg_{3}+\left(1+\mu \right)}<\upsilon \text{​}\right]+\left\{1-F_{g_{4}}\left(g_{0}\right)\right\}\times \mathrm{Pr}\left[g_{1}\geq \theta_{1},\frac{\left(1-\alpha \right)\gamma Xg_{3}}{1+\mu }<\upsilon \text{​}\right]\\ & =1-e^{-\lambda_{1}\theta_{1}}+\left(1-e^{-\lambda_{4}\theta_{0}}\right)\times \underset{\mathrm{Pr}3}{\underset{︸}{\mathrm{Pr}\left[X\geq \rho \eta \theta_{1}+\psi ,\;\gamma \left(1-\alpha -\upsilon \alpha \right)Xg_{3}<\upsilon \left(1+\mu \right)\;\right]}}\\ & \;+e^{-\lambda_{4}\theta_{0}}\times \underset{\mathrm{Pr}4}{\underset{︸}{\mathrm{Pr}\left[X\geq \rho \eta \theta_{1}+\psi ,\;\gamma \left(1-\alpha \right)Xg_{3}<\upsilon \left(1+\mu \right)\;\right]}} \end{array}$$

where the values of *θ*_0_ and *θ*_1_ are defined in (27).

When (1−α−υα) in the last equation of (46) satisfies (1−α−υα)≤0 or υ≥(1−α)/α, Pr3 in (46) is reduced to

(47) $$\mathrm{Pr}3=\mathrm{Pr}\left[X\geq \rho \eta \theta_{1}+\psi \right]=\mathrm{Pr}\left[g_{1}\geq \theta_{1}\right]=1-F_{g_{1}}\left(\theta_{1}\right)=e^{-\lambda_{1}\theta_{1}}$$

When υ<(1−α)/α, Pr3 is obtained as

(48) $$\begin{array}{ll} \mathrm{Pr}3 & =\mathrm{Pr}\left[X\geq \rho \eta \theta_{1}+\psi ,\;g_{3}<\frac{\upsilon \left(1+\mu \right)}{\gamma \left(1-\alpha -\upsilon \alpha \right)X}\;\right]\\ & =\int_{\rho \eta \theta_{1}+\psi }^{\infty }f_{X}\left(t\right)\times F_{g_{3}}\left(\frac{\upsilon \left(1+\mu \right)}{\gamma \left(1-\alpha -\upsilon \alpha \right)t}\right)dt\;\\ & =\int_{\rho \eta \theta_{1}+\psi }^{\infty }\frac{\lambda_{1}}{\rho \eta }\times e^{-\lambda_{1}\left(\frac{t-\psi }{\rho \eta }\right)}\times \left\{1-e^{-\lambda_{3}\frac{\upsilon \left(1+\mu \right)}{\gamma \left(1-\alpha -\upsilon \alpha \right)t}}\right\}dt\\ & =e^{-\lambda_{1}\theta_{1}}+\frac{\lambda_{1}e^{\lambda_{1}\;\psi /\left(\rho \eta \right)}}{\rho \eta }\times \int_{0}^{\rho \eta \theta_{1}+\psi }e^{-\frac{\lambda_{1}t}{\rho \eta }-\frac{\lambda_{3}\upsilon \left(1+\mu \right)}{\gamma \left(1-\alpha -\upsilon \alpha \right)t}}dt\\ & -2e^{\lambda_{1}\;\psi /\left(\rho \eta \right)}\sqrt{\frac{\lambda_{1}\lambda_{3}\upsilon \left(1+\mu \right)}{\gamma \rho \eta \left(1-\alpha -\upsilon \alpha \right)}}\times K_{1}\left(2\sqrt{\frac{\lambda_{1}\lambda_{3}\upsilon \left(1+\mu \right)}{\gamma \rho \eta \left(1-\alpha -\upsilon \alpha \right)}}\right) \end{array}$$

Similarly, Pr4 in (46) is rewritten as

(49) $$\begin{array}{l} \mathrm{Pr}4=\mathrm{Pr}\left[X\geq \rho \eta \theta_{1}+\psi ,\;g_{3}<\frac{\upsilon \left(1+\mu \right)}{\gamma \left(1-\alpha \right)X}\;\right]=\int_{\rho \eta \theta_{1}+\psi }^{\infty }f_{X}\left(t\right)\times F_{g_{3}}\left(\frac{\upsilon \left(1+\mu \right)}{\gamma \left(1-\alpha \right)t}\right)dt\\ =e^{-\lambda_{1}\theta_{1}}+\frac{\lambda_{1}e^{\lambda_{1}\psi /\left(\rho \eta \right)}}{\rho \eta }\int_{0}^{\rho \eta \theta_{1}+\psi }e^{-\frac{\lambda_{1}t}{\rho \eta }-\frac{\lambda_{3}\upsilon \left(1+\mu \right)}{\gamma \left(1-\alpha \right)t}}dt-2e^{\lambda_{1}\psi /\left(\rho \eta \right)}\sqrt{\frac{\lambda_{1}\lambda_{3}\upsilon \left(1+\mu \right)}{\gamma \rho \eta \left(1-\alpha \right)}}\times K_{1}\left(2\sqrt{\frac{\lambda_{1}\lambda_{3}\upsilon \left(1+\mu \right)}{\gamma \rho \eta \left(1-\alpha \right)}}\right) \end{array}$$

With (47), (48) and (49), the outage probability $P_{SN}^{cc}$ in (46) can be described as

(50) $$P_{SN}^{cc}=1-e^{-\lambda_{4}\theta_{0}}\times \left\{2e^{\lambda_{1}\psi /\left(\rho \eta \right)}\sqrt{\frac{\lambda_{1}\lambda_{3}\upsilon \left(1+\mu \right)}{\gamma \rho \eta \left(1-\alpha \right)}}\times K_{1}\left(2\sqrt{\frac{\lambda_{1}\lambda_{3}\upsilon \left(1+\mu \right)}{\gamma \rho \eta \left(1-\alpha \right)}}\right)-\frac{\lambda_{1}e^{\lambda_{1}\psi /\left(\rho \eta \right)}}{\rho \eta }\int_{0}^{\rho \eta \theta_{1}+\psi }e^{-\frac{\lambda_{1}t}{\rho \eta }-\frac{\lambda_{3}\upsilon \left(1+\mu \right)}{\gamma \left(1-\alpha \right)t}}dt\right\}$$

when υ≥(1−α)/α and

(51) $$\begin{array}{l} P_{SN}^{cc}=1-\left(1-e^{-\lambda_{4}\theta_{0}}\right)\times \left\{2e^{\lambda_{1}\psi /\left(\rho \eta \right)}\sqrt{\frac{\lambda_{1}\lambda_{3}\upsilon \left(1+\mu \right)}{\gamma \rho \eta \left(1-\alpha -\upsilon \alpha \right)}}\times K_{1}\left(2\sqrt{\frac{\lambda_{1}\lambda_{3}\upsilon \left(1+\mu \right)}{\gamma \rho \eta \left(1-\alpha -\upsilon \alpha \right)}}\right)-\frac{\lambda_{1}e^{\lambda_{1}\psi /\left(\rho \eta \right)}}{\rho \eta }\int_{0}^{\rho \eta \theta_{1}+\psi }e^{-\frac{\lambda_{1}t}{\rho \eta }-\frac{\lambda_{3}\upsilon \left(1+\mu \right)}{\gamma \left(1-\alpha -\upsilon \alpha \right)t}}dt\right\}\\ \;-e^{-\lambda_{4}\theta_{0}}\times \left\{2e^{\lambda_{1}\psi /\left(\rho \eta \right)}\sqrt{\frac{\lambda_{1}\lambda_{3}\upsilon \left(1+\mu \right)}{\gamma \rho \eta \left(1-\alpha \right)}}\times K_{1}\left(2\sqrt{\frac{\lambda_{1}\lambda_{3}\upsilon \left(1+\mu \right)}{\gamma \rho \eta \left(1-\alpha \right)}}\right)-\frac{\lambda_{1}e^{\lambda_{1}\psi /\left(\rho \eta \right)}}{\rho \eta }\int_{0}^{\rho \eta \theta_{1}+\psi }e^{-\frac{\lambda_{1}t}{\rho \eta }-\frac{\lambda_{3}\upsilon \left(1+\mu \right)}{\gamma \left(1-\alpha \right)t}}dt\right\} \end{array}$$

when υ<(1−α)/α.

Analytical expressions for the outage probabilities of the PN in (27) and the SN in (47) are readily used for evaluation, whereas the mathematical expressions for the PN in (37) and (38) and the SN in (50) and (51), require numerical analysis for evaluation.

## 4. Numerical Analysis and Simulation Results

Common simulation parameters are given in Table 1. The coordinates of the nodes are set at (0, 0) for the PT, (1, 0) for the PR, (*x*_1_, *y*_1_) for the ST, and (*x*_2_, *y*_2_) for the SR, where $0<x_{1},\;x_{2}<1$, so that the ST and the SR are located between the PT and the PR. The distances between the pairs of nodes are given as *d*_0_ = 1, $d_{1}=\sqrt{x_{1}^{2}+y_{1}^{2}}$, $d_{2}=\sqrt{{\left(1-x_{1}\right)}^{2}+y_{1}^{2}}$, $d_{3}=\sqrt{{\left(x_{2}-x_{1}\right)}^{2}+{\left(y_{2}-y_{1}\right)}^{2}}$, and $d_{4}=\sqrt{x_{2}^{2}+y_{2}^{2}}$. The path-loss exponent *β* is set at a typical value of ‘3’, and the energy conversion efficiency η is set to a constant value, 0.9 for our work, as in [12,14,16]. Equations (27), (37) and (38) are used for evaluation of the outage probability of the PN and (46), (47), (50) and (51) are adopted for the outage probability of the SN. Results of the Monte-Carlo simulation with (28) and the second equation of (27) (for the PN with RVs *g*_0_, *g*_1_, and *g*_2_) and the Monte-Carlo simulation of (46) (for the SN with RVs *g*_1_, *g*_3_, and *g*_4_) are denoted ‘Simu’ in the figures. The marginally optimal value $\rho_{PN,opt}$ and the jointly optimal value $\rho_{PN,jopt}$ are found from (27), (37) and (38) for the given system parameter(s). The fractional constant *Ψ*, representing the small internal power of the ST needed to transmit the power superposed codes, is set at 0.1 (in the range of *Ψ* values in [16]), unless otherwise stated.

### 4.1. Validation of Numerical Results

**Figure 2:**
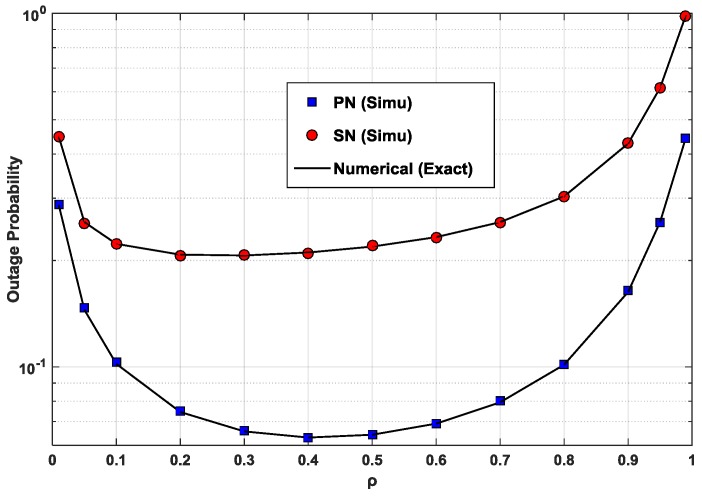
Outage probabilities of the PN and the SN in the CC scheme as a function of *ρ* when *P*/*N*_0_ = 10 dB, *x*_1_ = *x*_2_ = 0.5, *y*_1_ = 0.1, and *y*_2_ = 0.3.

In order to determine the accuracy of the numerical evaluation, a comparison between the numerical results and the simulation results is given in Figure 2. Figure 2 presents the outage probabilities of the PN and the SN as a function of *ρ* in the CC scheme. The SN is located on the median line of the PT–PR line segment. As can be seen in Figure 2, the two sets of results from different evaluation methods are in good agreement. The outage probability of the PN reaches its smallest value when *ρ* has the optimal value $\rho_{PN,opt}$ ≈ 0.4 for the given *α* = 0.9. Similarly, the outage probability is lowest with $\rho_{PN,opt}$ ≈ 0.2 for the SN. Due to the majority of the power being allocated to *x_p_*, the outage probability of the PN is significantly lower than that of the SN. These optimal values of $\rho_{PN,opt}$ and $\rho_{SN,opt}$ can be obtained exactly using the golden section search (GSS) method [36], along with a small tolerance parameter, (e.g., $\varepsilon =10^{-5}$). When the value of *ρ* is smaller than the optimal value $\rho_{PN,opt},$ the low level of harvested energy $E_{PT-ST}^{h}$ in (5) causes the signal level of the superposed codes in (15), received by the PR, to be comparatively low. The low SINR at the PR in (19) causes a higher outage probability of the PN. On the other hand, when the value of *ρ* is higher than $\rho_{PN,opt},$ the SNR of the primary signal in (6) becomes small, causing a smaller *R_AST_* in (25). The smaller *R_AST_* increases the outage probability of the PN in (22). Similarly, a value of *ρ* smaller than the optimal value $\rho_{SN,opt}$ is adverse to the signal level at the SR, according to (14) and (16); and a value of *ρ* higher than $\rho_{SN,opt}$, which causes a smaller *R_AST_* in (25), significantly increases the first term in (42). This is a marginal probability term and is in contrast to the joint probability terms in (22) for the PN. As suggested in Figure 2, the typically considered high power-sharing coefficients (e.g., most of the power allocated for the primary signal *x_p_* in the power superposition coding) allow ranges of power splitting ratios that provide relatively small outage probabilities for the PN and SN.

### 4.2. Jointly Optimal Power Allocation for PN and Range of Power Sharing Coefficient for Minimum PN Performance

**Figure 3:**
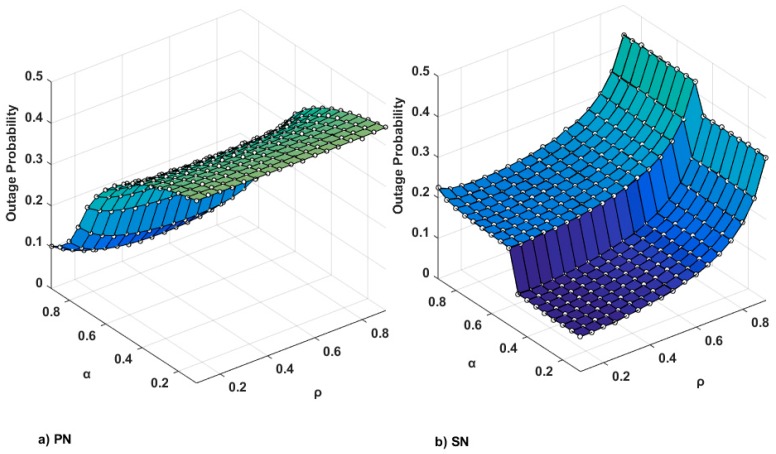
Outage probabilities of the PN and the SN as functions of *ρ* and *α* when *P*/*N*_0_ = 10 dB, *x*_1_ = *x*_2_ = 0.5, *y*_1_ = 0.1, and *y*_2_ = 0.3.

Figure 3 shows the outage probabilities of the PN and the SN as functions of *ρ* and *α*. The ranges of *ρ* and *α* are between 0.1 and 0.9. Symbols indicate the simulation results, while curves represent the numerical results. The outage probability of the PN is the lowest at about 0.06, when $\alpha_{PN,jopt}=0.9$ and $\rho_{PN,jopt}$ = 0.4; while the outage probability of the SN is the lowest at about 0.09, when $\alpha_{SN,jopt}=\rho_{SN,jopt}=0.1$. With the typically considered range of *α*, 0.8 ≤ *α* ≤ 0.9, the outage probability of the SN is at best 0.2. As seen in Figure 3, there is a range of power splitting ratio *ρ* that provides relatively small outage probabilities of the PN and the SN for each *α*. The outage probability of the SN with fixed *ρ*, gradually decreases with decrease of α. Unlike the case shown in Figure 3a for the PN, there is an abrupt decrement of the outage probability with the SN at *α* = 0.5. This is because of the rapid decrease of the probability Pr3 in (46). The value *α* = 0.5 is a root of (1−α−υα)=0, where $\upsilon =2^{2R_{s}}-1=1$, with *R_s_* = 0.5 (bits/s/Hz). When the target rate *R_s_* is increased, a sudden drop to lower α will be observed. At *α* ≈ 0.7 and *ρ* ≤ 0.5, the outage probability of the PN is close to that of the SN.

**Figure 4:**
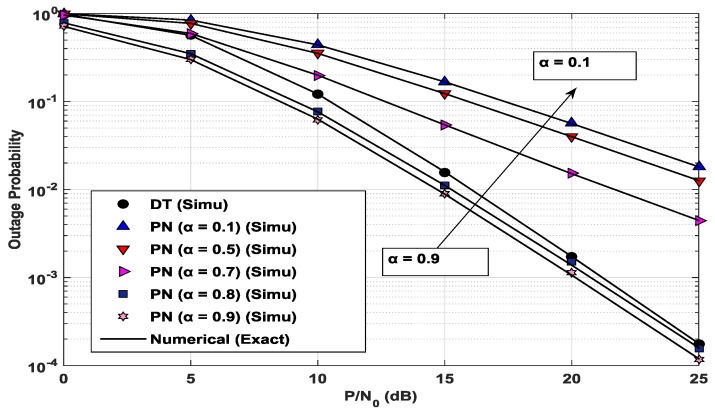
Outage probability of the PN in the CC scheme and in the DT scheme as a function of *P*/*N*_0_ when *x*_1_ = *x*_2_ = 0.5, *y*_1_ = 0.1, *y*_2_ = 0.3, and *α* = 0.1, 0.5, 0.7, 0.8, 0.9.

Figure 4 shows the outage probability of the PN with the CC scheme and the DT scheme, according to the SNR *γ* = *P/N*_0_, over different α values. It is noted that a larger α value indicates more power for the *x_p_* in power superposition coding. The power splitting ratio *ρ* is set at $\rho_{PN,opt}$ for each combination of SNR and α. This optimal value of $\rho_{PN,opt}$ is also obtained by the GSS method, using (27), (37) and (38), with the tolerance parameter $\varepsilon =10^{-5}.$ The outage probability of the DT scheme is evaluated using (41). The outage probability of the PN decreases when α increases, and the curve of the outage probability shows its lowest values when α is at its largest (i.e., *α* = 0.9). It can be seen that the outage performance of the PN with the CC scheme is better than that of the DT scheme, when the ST allocates a large fraction of the transmitted power (i.e., *α* ≥ 0.8), to the primary signal *x_p_*. It should be noted that the PN can achieve lower outage probability owing to the relaying by the ST, and the SN is able to take the opportunity to transmit its own data with somewhat higher outage probability.

**Figure 5:**
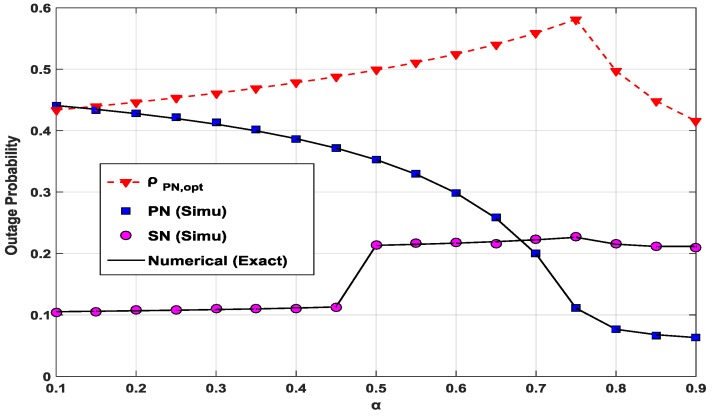
Outage probabilities and optimal power splitting ratio $\rho_{PN,opt}$ according to *α* when *P*/*N*_0_ = 10 dB, *x*_1_ = *x*_2_ = 0.5, *y*_1_ = 0.1 and *y*_2_ = 0.3.

Figure 5 shows the optimal power splitting ratio $\rho_{PN,opt}$ of the PN for each *α* and the consequent outage probabilities of the PN and SN with the two power parameters. It is noted that, with the CC scheme, the outage probability of the PN is more critical than that of the SN. The outage probability of the PN monotonically decreases when α increases. The range of $\rho_{PN,opt}$ over the variation of *α*, 0.1 < *α* ≤ 0.9, is between 0.42 and 0.58.

### 4.3. Impact of Other System Parameters on the Outage Probabilities of PN and SN

**Figure 6:**
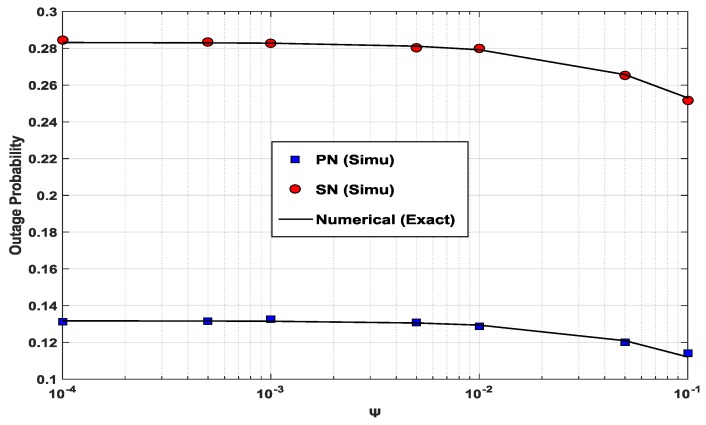
Outage probabilities of PN and SN as a function of *Ψ* when *P*/*N*_0_ = 10(dB), *α* = 0.9, *x*_1_ = *x*_2_ = 0.5, *y*_1_ = 0.1 and *y*_2_ = 0.3.

Figure 6 presents the outage probabilities of the PN and the SN as a function of *Ψ*. The range of the value of *Ψ* for the figure is between 10^−4^ and 10^−1^. The range of the value of *Ψ* here, overlaps well with those of [16] and [18]. The power splitting ratio *ρ* is set at $\rho_{PN,opt}$ for each *Ψ* value. The outage probabilities of the PN and the SN are seen to decrease slightly as the value of *Ψ* increases. Slight decrements of the outage probabilities of the PN and the SN are accounted for by the normalized distance, which is considered for channel gain *g*_1_ in (14). The value of the term $\rho \eta g_{1}$ in (14) is comparable to the *Ψ* value, so the *Ψ* value alone does not have a significant influence on the outage probabilities. Nonetheless, a higher *Ψ* value, which makes the transmit power of the ST become larger, causes lower outage probabilities for the PN and the SN.

**Figure 7:**
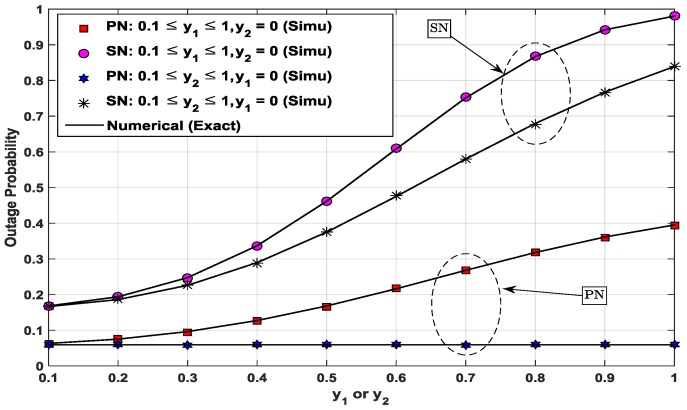
Outage probabilities of PN and SN as a function of *y*_1_ or *y*_2_ when *P*/*N*_0_ = 10 dB, *α* = 0.9, and *x*_1_ = *x*_2_ = 0.5.

Figure 7 shows the variations of the outage probabilities of the PN and the SN when either ST or SR changes its location. The changed location of the ST or the SR affects the relevant values of $\lambda_{i}\;(=d_{i}^{\beta })$ and $g_{i}$ in the pertinent equations (e.g., 27, 37 and 38) for the outage probability of the PN. The power splitting ratio is set to the optimal value $\rho_{PN,opt}$ for each combination of locations of the ST and the SR. This figure is to show the effect of the locations of the ST and the SR on the performance of the PN and the SN. First, the ST is fixed at the center (0.5, *y*_1_ = 0) and the SR at (0.5, *y*_2_) moves along the median line of the PT–PR line segment. Thus, the vertical axis indicates *y*_2_ at this time. As can be seen in the figure, the outage probability of the PN does not change when the ST is fixed at *y*_1_ = 0, whereas the outage probability of the SN increases due to the SR moving away from the ST. The outage performance of the PN is independent of the location of the SR. On the other hand, when the SR moves away from the PT and the ST, the SR suffers performance degradation, because of reduced SNR over longer distance: in decoding and in cancelling the primary signal *x_p_*, and decoding the signal *x_s_*. Second, the SR is set at (0.5, *y*_2_ = 0) and the ST is allowed to move along the median line of the PT–PR line segment, also from *y*_1_ = 0.1 to *y*_1_ = 1. The outage probabilities of the PN and the SN simultaneously increase because the level of the received signal at the ST becomes lower.

The dependency of network environments can be accounted for by different path-loss exponents (i.e., *β* ≠ 3). When *β* increases, the channel gains will decrease because $E\left[g_{i}\right]=1/\lambda_{i}=d_{i}^{-\beta }.$ The decreased channel gains over the wireless links under consideration, subsequently increase the outage probabilities of the PN and the SN, as estimated using (27), (37) and (38) for the PN and using (46), (47), (50) and (51) for the SN. If *β* decreases, the outage probabilities of the PN and the SN would also be expected to decrease. The impact of the energy conversion efficiency *η* in (5) and the value of *µ*, which describes the noise variance of the RFBCU, can be explained as follows. A larger value of *η* leads to more harvested energy, as can be determined from (5) and this causes the larger transmit power of the ST in (14). Hence, the SINRs at the PR and the SR become larger and, subsequently, the outage probabilities of the PN and the SN become lower. On the other hand, a larger µ value incurs a higher level of composite noise and causes higher outage probabilities of the PN and the SN.

## 5. Conclusions

A novel decode-and-forward type cooperative communication scheme based on energy harvesting and power superposition coding was investigated. At the same time, the ST adopts power splitting circuits to charge its internal battery and to decode the primary signal. A system model was established and analytical or mathematical expressions for performance evaluation were derived in terms of system parameters that included the power splitting ratio and power sharing coefficient. The system performance of the PN and the SN was evaluated in a twofold manner by numerical analysis and Monte-Carlo simulation. Our findings for the system parameters considered are as follows. The jointly optimal power splitting ratio and power sharing coefficient needed to achieve the minimum outage probability of the PN are different from the values needed for the minimum outage probability of the SN. The outage probability of the PN is lowest at about 0.06, when the power sharing coefficient is 0.9 and the power splitting ratio is 0.4; while the outage probability of the SN is lowest at about 0.09, when the power sharing coefficient and the power splitting ratio are both 0.1. A high power splitting ratio (e.g., an increased fraction for battery charging) is adverse to achieving low outage probabilities of the PN and the SN. The typically considered high power-sharing coefficients, (e.g., most of the power is allocated for the primary signal in the power superposition coding), allow a range of power splitting ratios that provide relatively small outage probabilities for the PN and SN.

## Figures and Tables

**Table 1:** Simulation parameters.

Parameter	Value
Power splitting ratio *ρ*	0 < *ρ* < 1
Power sharing coefficient *α*	0 < *α* < 1
Target rate *R_T_*	1 (bits/s/Hz)
Target rate *R_s_*	0.5 (bits/s/Hz)
Path-loss exponent *β*	3
Energy conversion efficiency *η*	0.9
Fractional constant *Ψ* for power provided by battery	0.1
Noise variance parameter *µ*	1

## Appendix A. Proof of Lemma 1

The joint CDF of RV *Y* and RV $g_{1}$ is expressed as

(A1) $$\begin{array}{ll} \mathrm{Pr}\left[Y<y,\;g_{1}\geq \theta_{1}\right] & =\mathrm{Pr}\left[\frac{\alpha \gamma Xg_{2}}{\left(1-\alpha \right)\gamma Xg_{2}+\left(1+\mu \right)}<y,\;g_{1}\geq \theta_{1}\right]\\ & =\mathrm{Pr}\left[\gamma \left[\alpha -y\left(1-\alpha \right)\right]g_{2}X<\left(1+\mu \right)y,X\geq \rho \eta \theta_{1}+\psi \right] \end{array}$$

Case 1: α−y(1−α)≤0 or y≥α/(1−α)

The expression *γ*[*α*-*y*(1-*α*)]*g*_2_*X* is always correct with positive *y*, so (A1) becomes

(A2) $$\mathrm{Pr}\left[X\geq \rho \eta \theta_{1}+\psi \right]=\mathrm{Pr}\left[g_{1}\geq \theta_{1}\right]=1-F_{g_{1}}\left(\theta_{1}\right)=e^{-\lambda_{1}\theta_{1}}$$

Case 2: y<α/(1−α)

(A3) $$\mathrm{Pr}\left[Y<y,\;g_{1}\geq \theta_{1}\right]=\mathrm{Pr}\left[X\geq \rho \eta \theta_{1}+\psi ,g_{2}<\;\underset{\phi \left(y\right)}{\underset{︸}{\frac{\left(1+\mu \right)y}{\gamma \left[\alpha -y\left(1-\alpha \right)\right]}}}\times \frac{1}{X}\right]=\int_{\rho \eta \theta_{1}+\psi }^{\infty }f_{X}\left(t\right)\times F_{g_{2}}\left(\frac{\phi \left(y\right)}{t}\right)dt$$

Substitution of the pdf of *X* in (30) for (A3) gives

(A4) $$\begin{array}{ll} \int_{\rho \eta \theta_{1}+\psi }^{\infty }f_{X}\left(t\right)\times F_{g_{2}}\left(\frac{\phi \left(y\right)}{t}\right)dt & =\int_{\rho \eta \theta_{1}+\psi }^{\infty }\frac{\lambda_{1}}{\rho \eta }e^{-\lambda_{1}\left(\frac{t-\psi }{\rho \eta }\right)}\times \left(1-e^{-\lambda_{2}\frac{\phi \left(y\right)}{t}}\right)dt\\ & =e^{-\lambda_{1}\theta_{1}}-\frac{\lambda_{1}}{\rho \eta }e^{\lambda_{1}\psi /\left(\rho \eta \right)}\times \left\{\int_{0}^{\infty }e^{\frac{-\lambda_{1}t}{\rho \eta }-\frac{\lambda_{2}\phi \left(y\right)}{t}}dt-\int_{0}^{\rho \eta \theta_{1}+\psi }e^{\frac{-\lambda_{1}t}{\rho \eta }-\frac{\lambda_{2}\phi \left(y\right)}{t}}dt\right\}\\ & =e^{-\lambda_{1}\theta_{1}}-2e^{\lambda_{1}\psi /\left(\rho \eta \right)}\sqrt{\frac{\lambda_{1}\lambda_{2}\phi \left(y\right)}{\rho \eta }}\;K_{1}\left(2\sqrt{\frac{\lambda_{1}\lambda_{2}\phi \left(y\right)}{\rho \eta }}\right)+\frac{\lambda_{1}}{\rho \eta }e^{\lambda_{1}\psi /\left(\rho \eta \right)}\int_{0}^{\rho \eta \theta_{1}+\psi }e^{\frac{-\lambda_{1}t}{\rho \eta }-\frac{\lambda_{2}\phi \left(y\right)}{t}}dt \end{array}$$

By (A2) and (A4), Lemma 1 is proved.

## Appendix B. Proof of Theorem 1

From Lemma 1, Pr2 in (36) can be rewritten as

(A5) $$\mathrm{Pr}2=\underset{\mathrm{Pr}21}{\underset{︸}{\int_{0}^{\tau -\alpha /\left(1-\alpha \right)}f_{\gamma_{PR}}\left(w\right)\times \mathrm{Pr}\left[X\geq \rho \eta \theta_{1}+\psi ,\;Y<(\tau -w)\right]dw}}+\underset{\mathrm{Pr}22}{\underset{︸}{\int_{\tau -\alpha /\left(1-\alpha \right)}^{\tau }f_{\gamma_{PR}}\left(w\right)\times \mathrm{Pr}\left[X\geq \rho \eta \theta_{1}+\psi ,\;Y<(\tau -w)\right]dw}}$$

Pr21 in (A5) is obtained as

(A6) $$\begin{array}{ll} \mathrm{Pr}21 & =\int_{0}^{\tau -\alpha /\left(1-\alpha \right)}e^{-\lambda_{1}\theta_{1}}\times f_{\gamma_{PR}}\left(w\right)dw\\ & =\left\{ \begin{array}{l} 0\;,\;\tau <\alpha /\left(1-\alpha \right)\\ e^{-\lambda_{1}\theta_{1}}\times F_{\gamma_{PR}}\left(\tau -\alpha /\left(1-\alpha \right)\right),\;\tau \geq \alpha /\left(1-\alpha \right) \end{array}\right.\\ & =\left\{ \begin{array}{l} 0\;,\;\tau <\alpha /\left(1-\alpha \right)\\ e^{-\lambda_{1}\theta_{1}}\times \left(1-e^{-\lambda_{0}\left(1+\mu \right)\left(\tau -\alpha /\left(1-\alpha \right)\right)/\gamma }\right),\;\tau \geq \alpha /\left(1-\alpha \right) \end{array}\right. \end{array}$$

where $F_{\gamma_{PR}}\left(x\right)$ is obtained from (31).

Pr22 in (A5) can be considered in two cases.

Case 1: τ<α/(1−α)

(A7) $$\mathrm{Pr}22=\int_{0}^{\tau }f_{\gamma_{PR}}\left(w\right)\times \mathrm{Pr}\left[X\geq \rho \eta \theta_{1}+\psi ,\;Y<(\tau -w)\right]dw$$

Substitutions of (32) and (34) into (A7) lead to

(A8) $$\begin{array}{ll} \mathrm{Pr}22 & =\int_{0}^{\tau }\frac{\lambda_{0}\left(1+\mu \right)e^{-\lambda_{0}\left(1+\mu \right)w/\gamma }}{\gamma }\times \{e^{-\lambda_{1}\theta_{1}}-2e^{\lambda_{1}\psi /\left(\rho \eta \right)}\sqrt{\frac{\lambda_{1}\lambda_{2}\phi \left(\tau -w\right)}{\rho \eta }}\;\times K_{1}\left(2\sqrt{\frac{\lambda_{1}\lambda_{2}\phi \left(\tau -w\right)}{\rho \eta }}\right)\\ & +\left(\left(\lambda_{1}e^{\lambda_{1}\psi /\left(\rho \eta \right)}\right)/\left(\rho \eta \right)\right)\times \int_{0}^{\rho \eta \theta_{1}+\psi }e^{-\lambda_{1}t/\left(\rho \eta \right)-\lambda_{2}\phi \left(\tau -w\right)/t}dt\}dw \end{array}$$

Substituting (35) into (A8) with a change of variable y≜τ−w, Pr22 is obtained as

(A9) $$\begin{array}{ll} \mathrm{Pr}22= & e^{-\lambda_{1}\theta_{1}}\left(1-e^{-\lambda_{0}\left(1+\mu \right)\tau /\gamma }\right)-\frac{2\lambda_{0}\left(1+\mu \right)}{\gamma }\sqrt{\frac{\lambda_{1}\lambda_{2}\left(1+\mu \right)}{\rho \eta \gamma \left(1-\alpha \right)}}\times e^{\lambda_{1}\psi /\left(\rho \eta \right)-\lambda_{0}\left(1+\mu \right)\tau /\gamma }\\ & \times \int_{0}^{\tau }\sqrt{\frac{y}{\alpha /\left(1-\alpha \right)-y}}\times K_{1}\left(2\sqrt{\frac{\lambda_{1}\lambda_{2}\left(1+\mu \right)}{\rho \eta \gamma \left(1-\alpha \right)}}\times \sqrt{\frac{y}{\alpha /\left(1-\alpha \right)-y}}\right)\times e^{\lambda_{0}\left(1+\mu \right)y/\gamma }dy\\ & +\frac{\lambda_{0}\lambda_{1}\left(1+\mu \right)}{\rho \eta \gamma }\times e^{\lambda_{1}\psi /\left(\rho \eta \right)-\lambda_{0}\left(1+\mu \right)\tau /\gamma }\int_{0}^{\tau }\int_{0}^{\rho \eta \theta_{1}+\psi }e^{\lambda_{0}\left(1+\mu \right)y/\gamma -\lambda_{1}t/\left(\rho \eta \right)-\frac{\lambda_{2}\left(1+\mu \right)y}{\gamma \left(\alpha /\left(1-\alpha \right)-y\right)\left(1-\alpha \right)t}}dtdy \end{array}$$

Case 2: τ≥α/(1−α)

(A10) $$\mathrm{Pr}22=\int_{\tau -\alpha /\left(1-\alpha \right)}^{\tau }f_{\gamma_{PR}}\left(w\right)\times \mathrm{Pr}\left[X\geq \rho \eta \theta_{1}+\psi ,\;Y<(\tau -w)\right]dw$$

Substituting (32) and (34) into (A10) with a change of variable of y≜τ−w, Pr22 is obtained as

(A11) $$\begin{array}{ll} \mathrm{Pr}22= & e^{-\lambda_{1}\theta_{1}}\left(e^{-\lambda_{0}\left(1+\mu \right)\left(\tau -\alpha /\left(1-\alpha \right)\right)/\gamma }-e^{-\lambda_{0}\left(1+\mu \right)\tau /\gamma }\right)-\frac{2\lambda_{0}\left(1+\mu \right)}{\gamma }\sqrt{\frac{\lambda_{1}\lambda_{2}\left(1+\mu \right)}{\rho \eta \gamma \left(1-\alpha \right)}}\\ & \times e^{\lambda_{1}\psi /\left(\rho \eta \right)-\lambda_{0}\left(1+\mu \right)\tau /\gamma }\int_{0}^{\alpha /\left(1-\alpha \right)}\sqrt{\frac{y}{\alpha /\left(1-\alpha \right)-y}}\times K_{1}\left(2\sqrt{\frac{\lambda_{1}\lambda_{2}\left(1+\mu \right)}{\rho \eta \gamma \left(1-\alpha \right)}}\times \sqrt{\frac{y}{\alpha /\left(1-\alpha \right)-y}}\right)\times e^{\lambda_{0}\left(1+\mu \right)y/\gamma }dy\\ & +\frac{\lambda_{0}\lambda_{1}\left(1+\mu \right)}{\rho \eta \gamma }\times e^{\lambda_{1}\psi /\left(\rho \eta \right)-\lambda_{0}\left(1+\mu \right)\tau /\gamma }\int_{0}^{\alpha /\left(1-\alpha \right)}\int_{0}^{\rho \eta \theta_{1}+\psi }e^{\lambda_{0}\left(1+\mu \right)y/\gamma -\lambda_{1}t/\left(\rho \eta \right)-\frac{\lambda_{2}\left(1+\mu \right)y}{\gamma \left(\alpha /\left(1-\alpha \right)-y\right)\left(1-\alpha \right)t}}dtdy \end{array}$$

With (A6), (A9) and (A11), the proof of Theorem 1 is complete.
